# Preliminary evidence for the phosphodiesterase type-4 inhibitor, roflumilast, in ameliorating cognitive flexibility deficits in patients with schizophrenia

**DOI:** 10.1177/02698811211000778

**Published:** 2021-04-28

**Authors:** Nicholas R Livingston, Peter CT Hawkins, James Gilleen, Rong Ye, Lorena Valdearenas, Sukhi S Shergill, Mitul A Mehta

**Affiliations:** 1 Department of Neuroimaging, King’s College London, London, UK; 2 Department of Psychology, University of Roehampton, London, UK; 3 Department of Psychosis Studies, King’s College London, London, UK; 4 Department of Clinical Neurosciences, University of Cambridge, Cambridge, UK; 5 North Middlesex University Hospital, Barnet, Enfield and Haringey Mental Health NHS Trust, London, UK

**Keywords:** Schizophrenia, phosphodiesterase inhibitor, cognitive deficit, attentional set-shifting, functional magnetic resonance imaging

## Abstract

**Background:**

Cognitive flexibility deficits are present in patients with schizophrenia and are strong predictors of functional outcome but, as yet, have no pharmacological treatments.

**Aims:**

The purpose of this study was to investigate whether the phosphodiesterase type-4 inhibitor, roflumilast, can improve cognitive flexibility performance and functional brain activity in patients with schizophrenia.

**Methods:**

This was a within-subject, randomised, double-blind, placebo-controlled, three-period crossover study using a version of the Intradimensional/Extradimensional (ID/ED) task, optimised for functional magnetic resonance imaging (fMRI), in 10 patients with schizophrenia who were scanned after receiving placebo, 100 µg or 250 µg roflumilast for 8 consecutive days. Data from an additional fMRI ID/ED study of 18 healthy participants on placebo was included to contextualise the schizophrenia-related performance and activations. The fMRI analyses included *a priori* driven region of interest (ROI) analysis of the dorsal frontoparietal attention network.

**Results:**

Patients on placebo demonstrated broad deficits in task performance compared to the healthy comparison group, accompanied by preserved network activity for solution search, but reduced activity in left ventrolateral prefrontal cortex (VLPFC) and posterior parietal cortex for attentional set-shifting and reduced activity in left dorsolateral prefrontal cortex (DLPFC) for reversal learning. These ROI deficits were ameliorated by 250 µg roflumilast, whereas during solution search 100 µg roflumilast reduced activity in the left orbitofrontal cortex, right DLPFC and bilateral PPC, which was associated with an improvement in formation of attentional sets.

**Conclusions:**

The results suggest roflumilast has dose-dependent cognitive enhancing effects on the ID/ED task in patients with schizophrenia, and provides sufficient support for larger studies to test roflumilast’s role in improving cognitive flexibility deficits in this clinical population.

## Introduction

Schizophrenia is typically characterised by positive and negative symptoms (American Psychiatric Association, 2013), but patients also display cognitive deficits, for which there are no approved targeted pharmacological treatments. Developing treatments for cognitive deficits in schizophrenia is an important objective because they are severe (Mesholam-Gately et al., 2009), extend across several cognitive domains (Georgiades et al., 2017), persist through periods of remission of other symptoms, such as positive psychotic symptoms (Hughes et al., 2003), and are among the best predictors of functional outcome (Chang et al., 2016; Green, 1996).

One of the most pronounced cognitive deficits observed in schizophrenia is in the domain of attention, with meta-analyses reporting a large effect size (−0.8 to −1.4) of poorer performance in drug naïve (Fatouros-Bergman et al., 2014), first-episode (Mesholam-Gately et al., 2009; Zhang et al., 2019) and chronic (Bora et al., 2017) schizophrenia patients compared to healthy controls. In particular, the literature focuses on cognitive flexibility (Elvevag et al., 2012), first defined as an individual’s ability to modulate behaviour in response to environmental cues (Scott, 1962). An attentional set is developed when an individual learns that a specific element of a stimulus maintains relevance in different contexts, biasing the individual’s directed attention and inhibiting processing of irrelevant information (Rushworth et al., 2005). Attentional set-shifting tasks assess cognitive flexibility by evaluating how effectively individuals shift their attentional set in the presence of rule changes. One example includes the two-choice compound stimuli discrimination task called the Intradimensional/Extradimensional (ID/ED) task (Roberts et al., 1988). In this task, participants must select a target stimulus (e.g. a picture of a building or face) and then maintain responding to this target until there is a rule change, whereby the target stimuli will change. Following a rule change, there are two types of set-shift: within the same dimension, termed an intradimensional (ID) shift (e.g. from a building to a different building), or between dimensions, termed an extradimensional (ED) shift (e.g. building to face), with the latter typically requiring more trials to successfully solve (Roberts et al., 1988). This task has been validated in lesion studies (Dias et al., 1996) and neuroimaging studies (Hampshire and Owen, 2006), is sensitive to pharmacological modulation (Tait et al., 2014), and has been extensively used to examine set-shifting deficits in schizophrenia (Waltz, 2017).

Evidence as to the extent of ID/ED task deficits in patients with schizophrenia is mixed. The majority of studies report a deficit in performance (see Hilti et al., 2010 for an exception), differing in the task stage at which the deficit is observed (Murray et al., 2008; Pantelis et al., 1999, 2004). The literature shows a general increase in the severity of the deficit with length of illness. First-episode psychosis patients show a selective deficit in ED shifting (Hutton et al., 1998; Joyce et al., 2002; Murray et al., 2008; Pantelis et al., 2009) that is dependent on premorbid IQ (Ceaser et al., 2008), and this extends to other task components (e.g. ID shifting) with longer duration of illness (Joyce et al., 2002; Pantelis et al., 2004), as well as increasing severity of state-like symptoms (Pantelis et al., 2004, 2009).

In animal models of schizophrenia (such as phencyclidine (PCP), dizocilpine (MK-801) and ketamine models), impaired ED shift performance has been successfully recovered by a variety of receptor-targeting drugs, including atypical antipsychotics (Goetghebeur et al., 2009), serotonin receptor antagonists (Rodefer et al., 2008) and dopamine agonists (Fletcher et al., 2005). However, the effects of these receptor-targeting drugs might be limited to short-term use, as compensatory adaptive mechanisms of synaptic signalling could reduce their efficacy (Barak et al., 2011). Additionally, there are no receptor-targeting drug mechanisms that have successfully been translated from animal models to patients (Goetghebeur et al., 2014). More recent research has focused on drugs which indirectly target neurotransmitter systems by altering a secondary component of synaptic signalling, including phosphodiesterase (PDE) inhibitors (Duinen et al., 2015).

PDE inhibitors prevent the breakdown of cyclic adenosine monophosphate (cAMP), and this elevated cAMP can alter synaptic plasticity (Imanishi et al., 1997) and postsynaptic signalling (Song et al., 2013). Prefrontal cAMP concentration is related to the interaction of dopamine D1 and N-methyl-D-aspartate (NMDA) receptors (Pei et al., 2004), both of which are dysfunctional in schizophrenia (Balu, 2016; Goldman-Rakic et al., 2004), suggesting their association to cognitive deficits in this patient population could be due to reduced cAMP. Drugs which indirectly enhance NMDA receptor function (and consequently increase cAMP), such as sodium benzoate, have previously been shown to improve cognitive deficits in patients with schizophrenia (Lane et al., 2013; Lin et al., 2017), emphasising the potential utility of other drugs that elevate cAMP, such as PDE inhibitors. In particular, rolipram, a PDE type-4 (PDE4) inhibitor, has been shown to enhance cAMP signalling both in slices and *in vivo* (Bateup et al., 2008), as well as improving ED shift performance in rodent models of schizophrenia (Rodefer et al., 2012). However, in humans, rolipram has a relatively severe side-effect profile compared to other PDE4 inhibitors, such as roflumilast. Roflumilast, currently used to treat chronic obstructive pulmonary disorder, has demonstrable cognitive-enhancing effects, such as improving memory in rats (Jabaris et al., 2015; Nagakura et al., 2002; Vanmierlo et al., 2016), healthy human participants (Blokland et al., 2019; Van Duinen et al., 2018) and patients with schizophrenia (Gilleen et al., 2018). Additionally, its cognitive enhancing effects in patients with schizophrenia has been observed with electroencephalography (EEG; Gilleen et al., 2021).

On the basis of the prevalence of set-shifting deficits in schizophrenia, the deficit on ID/ED tasks in animal models and their recovery by other PDE4 inhibitors, roflumilast was tested here to see if it can improve impairments on the ID/ED task in patients with schizophrenia. Task-based functional magnetic resonance imaging (fMRI) was used as an intermediate measure as it is sensitive to process-specific modulation of brain activity, without relying on performance changes alone. The critical processes in the ID/ED task are correct responding (when the rule is known), searching (when the rule is not yet known), ID shifting (a set-shift within the same dimension), ED shifting (a set-shift between dimensions) and reversals (when the rule is changed by swapping the stimulus reward contingencies, as opposed to introducing a new set of stimuli). Neuroimaging studies to date have shown the dorsolateral prefrontal cortex (DLPFC) is activated in general searching (Hampshire and Owen, 2006), the ventrolateral prefrontal cortex (VLPFC; Konishi et al., 2002; Monchi et al., 2001; Rogers et al., 2000) and posterior parietal cortex (PPC; Asari et al., 2005) in ED shifting, whilst the orbitofrontal cortex (OFC) is activated for reversal events (Hampshire and Owen, 2006).

This study had three main aims. First, to replicate previous behavioural and imaging findings in the healthy comparison group to validate that the task fractionates attentional set-shifting as expected. Second, to measure behavioural performance and functional brain activity in patients with schizophrenia on placebo in order to better characterise the nature of their deficits. Third, to investigate the potential of roflumilast to improve attentional set-shifting deficits in patients with schizophrenia, by examining the changes in brain activity and behavioural performance within the different task components. We hypothesised that improved attentional set-shifting would be associated with reduction in errors during ED shifting, accompanied by modulation of activation within the lateral prefrontal cortex (PFC).

## Methods

### Study design

This study utilised two datasets from placebo-controlled drug studies employing the ID/ED task. The first dataset (Dataset 1) is the placebo group from a study in healthy participants. The second dataset (Dataset 2) comprises both placebo and roflumilast arms from a study in patients with schizophrenia. The inclusion criteria, exclusion criteria and participant demographics for both the previous studies are shown in Table 1.

**Table 1.** Table showing the relevant inclusion and exclusion criteria, as well as participant demographic information, for dataset 1 and 2.

	Dataset 1	Dataset 2
*Inclusion criteria*	45–70 years old	18–60 years old
	Right handed	Right handed
		DSM-V diagnosis of schizophrenia
		Stable dose of SGA for at least 2 months prior to screening
*Exclusion criteria*	History of psychiatric or neurological disorders	Failed drug screening
	Concurrent medication that affects the administration of atomoxetine	History of illicit drug or alcohol abuse within 6 months prior to screening
	Medical conditions that affect hepatic, renal or gastrointestinal function	Treatment with clozapine in last year
	Cardiac disorders	Unwilling to abstain from illicit drug or alcohol use throughout study
	Excessive use of nicotine (>5 cigarettes/day), caffeine (>400 mg/day) and alcohol (>28 units/week)	MRI contraindications
	Pre-menopausal women	Clinically significant neurological abnormalities
	MRI contraindications	
*Participant demographic – mean (SD)*		
Age, years	57.7 (8.3)	37.4 (7.9)
Gender (male:female)	10:8	6:4

*Clinical information – mean (SD)*		
Age of illness onset, years	N/A	31.3 (6.2)
Age of illness duration, months	N/A	152.3 (123.3)
Time on current SGA, months	N/A	71.1 (58.7)
Baseline PANSS total	N/A	63.7 (15.8)
Baseline PANSS positive	N/A	15.3 (3.8)
Baseline PANSS negative	N/A	16.3 (5.2)
Baseline PANSS general	N/A	32.1 (7.8)

DSM-V: Diagnostic and Statistical Manual of Mental Disorders, 5^th^ Edition; MRI: magnetic resonance imaging; PANSS: Positive and Negative Syndrome Scale; SD: standard deviation; SGA: second generation antipsychotic.

Dataset 1 includes data from a previous within-subject, randomised, double-blind, placebo-controlled study examining atomoxetine on the ID/ED task, and consisted of 19 healthy, right-handed, male and female participants aged 45–70 years old. One participant was excluded in this study due to poor task performance, prohibiting modelling of their imaging data and leaving 18 valid datasets. Only the placebo scans were used in the current study as a healthy comparison group. This data set was included for two reasons. First, due to the limited use of this task in the literature, it served as a replication of previous findings. Second, to allow us to understand if any drug effects in Dataset 2 were in the direction of normalisation. The study had King’s College London (KCL) College Research Ethics Committee (Psychiatry, Nursing and Midwifery Research Ethics Subcommittee) approval (HR-15/16-1964).

Dataset 2 includes data from a previous within-subject, randomised, double-blind, placebo-controlled, three-period crossover study. It investigated the effect of roflumilast on brain activity and task performance in patients with schizophrenia, with the ID/ED task a secondary endpoint (primary endpoints have been reported previously (Gilleen et al., 2018)). There were three arms to the study, in which the patients either took placebo, 100 µg or 250 µg roflumilast once a day, every day, for eight consecutive days. On the 8th day, the patients underwent an fMRI scan whilst performing the task. Patients were randomly allocated to one of three treatment sequences according to a Latin Square design (ABC, BCA or CAB), with a two-week wash-out period between arms. It recruited 21 right-handed, male and female patients with schizophrenia aged 18–60 years old, but six patients withdrew, and five datasets were excluded due to poor task performance (as determined by failure to successfully shift and learn more than one rule change), leaving 10 valid complete datasets included in this study. The study was pre-registered with clinicaltrials.gov (identifier: NCT02079844), and hospital ethics approvals were given by National Research Ethics Service (NRES) Committee South Central Berkshire (reference 12/SC/0443). The primary outcomes of the trial have been published elsewhere (Gilleen et al., 2018).

### Task design

**Figure 1.**
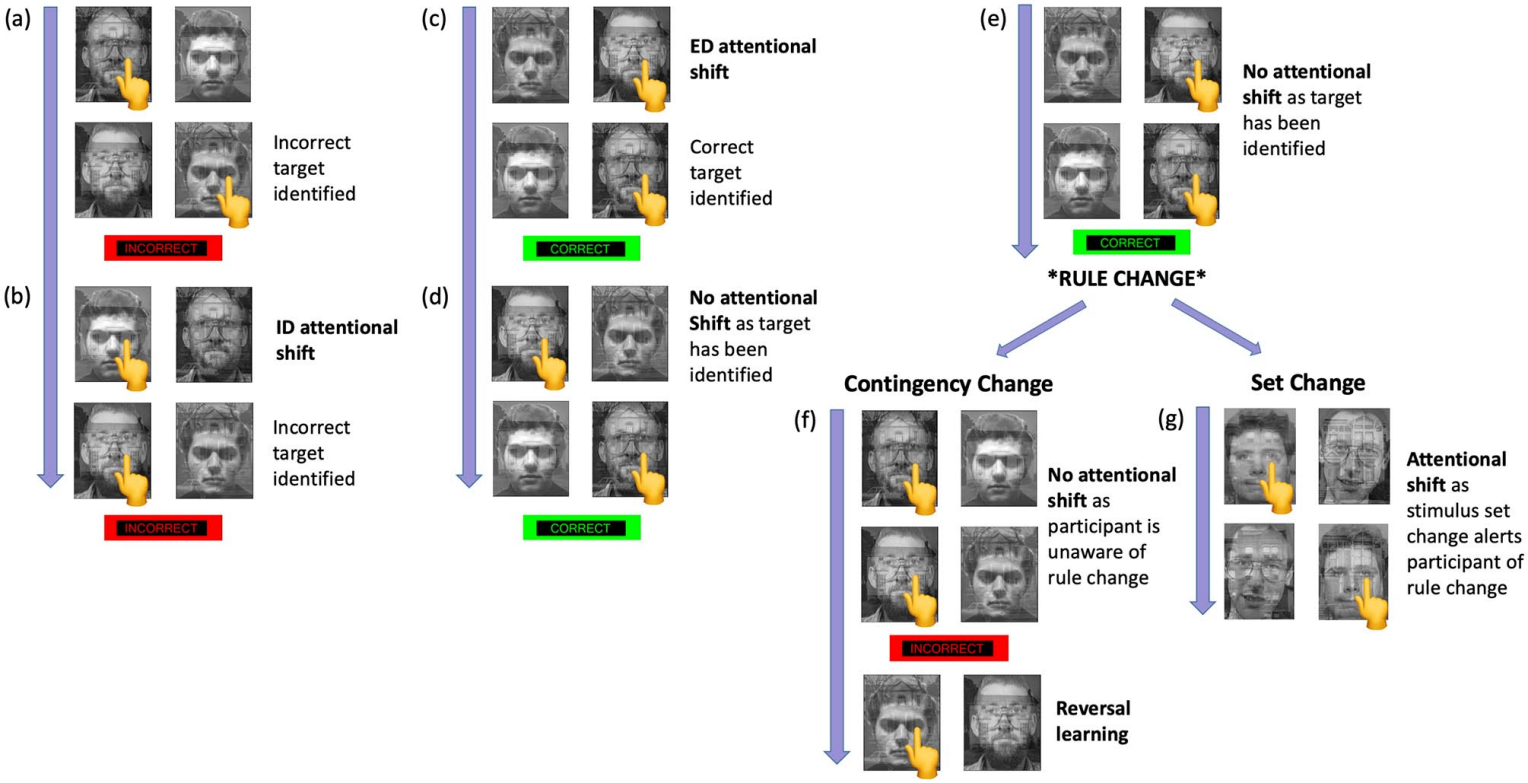
Schematic example of the Hampshire and Owen Intradimensional/Extradimensional (ID/ED) task. The participant identifies the target face/building from the two compound stimuli. They are given feedback after two responses. Example: (a) In the first of the couplet, the participant chooses to follow the left building, so selects the left compound stimuli. In the second of the couplet, the stimuli are presented again, but the pairing combination is swapped. The participant now chooses the right compound stimuli, as this contains the same building. This target stimulus is incorrect and the participant receives negative feedback. (b) The participant now engages in an intradimensional (ID) shift as they shift *within* dimensions and choose the other building, but this is also incorrect and the participant again receives negative feedback. (c) The participant now engages in an extradimensional (ED) shift, as they shift *between* dimensions and choose one of the faces. This is the correct target stimulus and the participant receives positive feedback, termed ‘early correct response’. (d) and (e) The participant follows the target stimulus for another two couplets, until the participant has reached the criteria of six correct responses in a row, termed ‘late correct response’. Following this, there is a rule change which can occur in two different ways, either through a contingency change or a set change. (f) Contingency change: here, the stimuli remain the same, but the stimulus-reward contingencies are swapped, as one of the other three stimuli is now the target. Therefore, the participant must engage in a reversal learning by inhibiting their response to the previously correct stimulus (which is now incorrect) and selecting another stimulus. (g) Set change: alternatively, brand new stimuli can be introduced, whereby the participant engages in an attentional shift and begins searching for the new target stimuli.

In this version of the ID/ED task (identical version used in both datasets), participants are required to find the target stimulus (either face or building) within two compound images, with each image (presented on the left or right of the screen) being composed of a face and a building (Figure 1; Hampshire and Owen, 2006). After choosing a compound image with a left or right button press, the stimuli are removed for 2 s before the stimuli are presented again, but now with the face-building combination swapped between the pairs. Assuming the participant selects the same target stimuli for each trial of the same couplet, this swapping of combinations allows the computer to determine which target the participant is following. Following the second response, the participant receives either ‘correct’ (in green) or ‘incorrect’ (in red) feedback, displayed on the screen for 1 s. Correct feedback was only given if the participant had correctly chosen the target stimulus on both responses. Following an incorrect response, participants must shift their attention to pick a new target stimulus either within the same dimension (face to face or building to building), termed ID shift, or between dimensions (face to building or building to face), termed ED shift. Following six consecutive correct responses, one of two possible events occurs. The first is termed a ‘set change’, whereby the participant is presented with new stimuli. The second is termed a ‘contingency change’, whereby the stimuli remain the same, but the target changes to one of the other three previous non-target stimuli. In this latter condition, the participant must engage in reversal learning, by inhibiting their response to the previously correct stimuli (which is now incorrect) and reversing the stimuli reward associations. Importantly, a rule change following either a contingency change or a set change can require either an ID or an ED shift, allowing for the fractionating of different cognitive components of the task. The number of errors made by participants whilst searching for the target were split into four categories, based upon the necessary dimension shift to identify the target (ID or ED) and the type of target change (set change or contingency change).

### Magnetic resonance imaging (MRI) data acquisition

Both studies used a 3.0 T GE scanner with a 32-channel head coil to collect functional data during the task (Dataset 1 repetition time (TR) = 2000 ms, echo time (TE) = 30 ms, field of view (FoV) = 240 mm, voxel size 3.75 × 3.75 × 3.3 mm, 39 slices; Dataset 2 TR = 2000 ms, TE = 30 ms, FoV = 211 mm, voxel size 3.3 × 3.3 × 3.3 mm, 39 slices) but also a high-resolution magnetisation prepared rapid acquisition gradient echo (MPRAGE) image for the registration and normalisation of the functional data (TR = 7312 ms, TE = 3.016 ms, FoV=270 mm, voxel size 1.05 × 1.05 × 1.2 mm, 196 slices, inversion time (TI) = 400 ms).

### Behavioural data analysis

Behavioural performance was analysed using Jamovi version 0.9.6.8 as described in previous studies (Hampshire and Owen, 2006; Williams-Gray et al., 2008). An error is denoted by selecting the incorrect stimulus for any given trial i.e. if the participant selected the wrong stimulus for both trials in a couplet, this would be two errors. Three general linear models (GLMs) were run examining errors made in the following groups: (a) healthy comparison group, with score as dependent variable, and with ‘shift’ (ED vs ID) and ‘target’ (set change vs contingency change) as factors, (b) patients with schizophrenia on placebo, in an identical GLM and (c) patients with schizophrenia on placebo vs the healthy comparison group, in the same GLM but with ‘group’ as an additional factor. To investigate drug effects on behavioural performance, data from patients with schizophrenia on placebo, 100 µg roflumilast and 250 µg roflumilast was fitted to a linear mixed-effects model, with ‘score’ as the dependent variable, and ‘shift’, ‘target’, ‘drug type’ and ‘drug order’ as factors, with ‘subject’ as the cluster variable. Post hoc analyses were conducted using *t*-tests (Bonferroni-corrected).

### MRI data analysis

Imaging data was preprocessed and modelled using SPM 12 (Wellcome Centre for Human Neuroimaging, UCL, UK) in MATLAB (R2014a; The Mathworks Inc., USA).

#### Preprocessing

The origin of all imaging data was reset to the anterior commissure in order to allow optimum co-registration. The T1-weighted images were segmented into grey matter (GM), white matter (WM) and cerebrospinal fluid (CSF), and a study-specific template was generated from the GM and WM maps using diffeomorphic anatomical registration through exponentiated lie algebra (DARTEL; Ashburner, 2007). The functional scans underwent slice timing correction, two-pass realignment and were co-registered to the relevant T1-weighted image. DARTEL flow fields were used to normalise the co-registered functional scans to Montreal Neurological Institute (MNI) space and an 8 mm full width at half maximum (FWHM) Gaussian kernel was used to smooth the data.

#### Event modelling

The blood oxygen level-dependent (BOLD) response was modelled to the onset times and durations of seven conditioned events. In four events, participants shifted their attention: ID shifts, ED shifts, set change and contingency change. It is important to note that whilst the behavioural data is measuring the number of errors for each type of required dimensional shift and type of target change, the imaging is modelled from participant-led attentional shifts whilst searching for the target. Previous authors have modelled each of these shifting events as the second response of each couplet (Chamberlain et al., 2008; Hampshire and Owen, 2006; Williams-Gray et al., 2008). However, the second response of the couplet may no longer contain the cognitive processes involved in attentional set-shifting, but rather other cognitive processes, such as working memory. Therefore, in this study, only the first in each pair of stimuli was modelled as these events. ID shift onset was the presentation of the first stimuli in a pair, with a duration up until the first button press when the participant’s selection involved a shift within the same dimension compared to the previous trial selection (e.g. building to building). ED shift was the same onset and duration, but defined as when the participant’s selection involved a shift between dimensions (e.g. building to face). Set change onset was from the presentation of a new set of stimuli, with duration up until the first button press. Contingency change onset was presentation of an image which preceded the first change of tactic after a rule change, until the button press denoting that change. Additional regressors were specified for known responses (correct selections made when the rule had been learnt, with onset as presentation of the first stimuli in the prospective third correctly selected pair up until first button press) and one each for positive and negative feedback (onset as correct/incorrect button press through to the presentation of the feedback).

#### Contrasts

Three contrasts were made at the first level: (a) searching for the target (known events > ID shifts + ED shifts + contingency change + set change), (b) attentional set-shifting (ED shifts > ID shifts) and (c) reversal learning (contingency change > set change).

#### Region of interest (ROI) analyses

**Figure 2.**
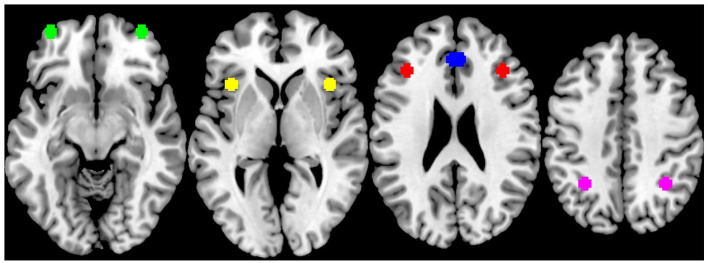
Visual representation of the 10 spherical regions of interest (ROIs) placed bilaterally across the dorsal frontoparietal attention network as taken from Hampshire and Owen (2006). From left to right, this includes the lateral orbitofrontal cortex (green), ventrolateral prefrontal cortex (yellow), dorsolateral prefrontal cortex (red), medial orbitofrontal cortex (blue) and posterior parietal cortex (pink).

ROI analysis was the primary method of analysis as studied previously on this task (Hampshire et al., 2006; Williams-Gray et al., 2008) due to *a priori* hypotheses around the regions identified in the literature. It was conducted bilaterally on the dorsal frontoparietal attention network, comprising of medial OFC, lateral OFC, VLPFC, DLPFC and PPC, using 10 spherical ROIs with a radius of 5 mm and the coordinates given in Hampshire and Owen (2006; Figure 2). The MNI coordinates for these ROIs were as follows: left medial OFC (*x* = −3, *y* = 37, *z* = −21), right medial OFC (*x* = 3, *y* = 37, *z* = −21), left lateral OFC (*x* = −36, *y* = 58, *z* = −12), right lateral OFC (*x* = 36, *y* = 58, *z* = −12), left VLPFC (*x* = −39, *y* = 20, *z* = 2), right VLPFC (*x* = 39, *y* = 20, *z* = 2), left DLPFC (*x* = −38, *y* = 30, *z* = 22), right DLPFC (*x* = 38, *y* = 30, *z* = 22), left PPC (*x* = −31, *y* = −53, *z* = 40) and right PPC (*x* = 34, *y* = −52, *z* = 41). The ROIs were created using the MARSeille Boîte A Région d’Intérêt (MarsBaR) toolbox in SPM (Brett et al., 2002) and the beta value for each ROI in each contrast for each dataset was extracted for statistical analysis. Before the main analysis, the data was assessed for the effects of age and tested for outliers. Since age differed between groups, we could not simply use age as a covariate (Miller et al., 2001). A correlation matrix was calculated between ROI beta values and age for the healthy comparison group and patients with schizophrenia on placebo across the three contrasts to test for age effects within each group and to understand if age explained the variation in ROI data. No significant correlations were found, implying there were no age effects on the ROI data. Outliers were identified using SPSS as values greater or smaller than 1.5× the interquartile range. Where the datasets contained outliers, non-parametric tests were used.

For the healthy comparison group and patients with schizophrenia on placebo, multiple one sample *t*-tests (against 0) were run on each of the ROIs for each of the three contrasts (false discovery rate (FDR) corrected *p*-value with discovery rate of *p* = 0.1) to observe which ROIs were significantly activated for each group. To investigate group differences in ROI activity, all three contrasts were analysed individually using a Kruskal-Wallis H test of analysis of variance (ANOVA). For these, the task contrast value within the ROI was the dependent variable, with group as the between-subjects factor. Post hoc analyses were conducted using Mann-Whitney *U* tests (FDR-corrected) due to outliers leading to non-homogeneity of variance between groups. The data from patients with schizophrenia on placebo, 100 µg and 250 µg roflumilast were analysed using a repeated measures ANOVA, with ‘ROI’ and ‘Drug’ as within-subject factors and ‘drug order’ as between-subjects factor. Post hoc analyses were conducted using paired *t*-tests (FDR-corrected).

## Results

### Behavioural results

#### Healthy comparison group

**Figure 3.**
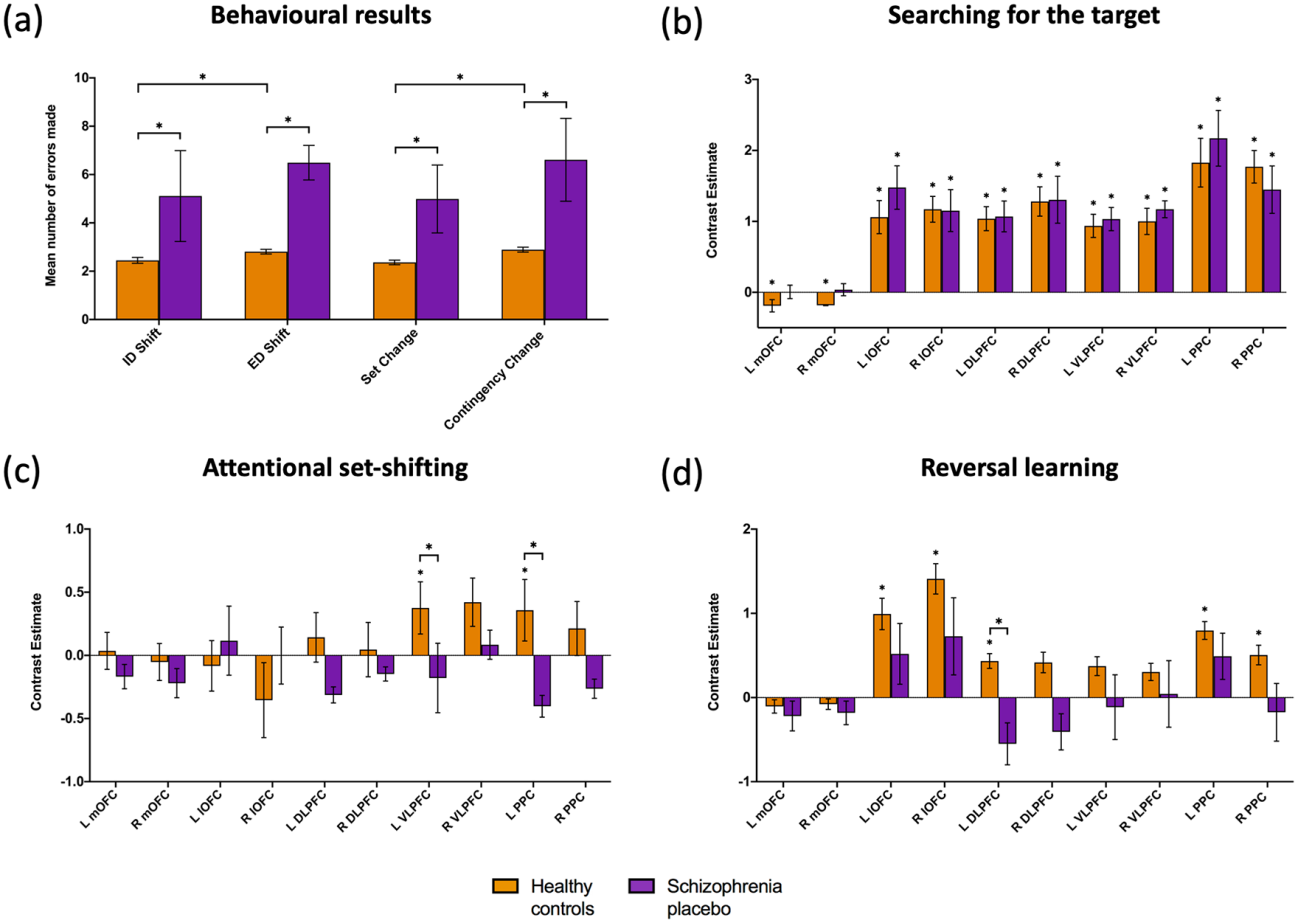
Behavioural and imaging results for the healthy comparison group and patients with schizophrenia on placebo: (a) bar chart showing mean number of errors made according to category type of dimensional shift (intradimensional (ID) and extradimensional (ED)) and target change (set change and contingency change); (b) bar chart showing beta values extracted from the 10 regions of interest (ROIs) for the searching > known contrast; (c) bar chart showing beta values extracted from the 10 ROIs for the ED shift > ID shift contrast; (d) bar chart showing beta values extracted from the 10 ROIs for the contingency change > set change contrast. Error bars represent standard error of the mean (SEM) and * denotes *p* < 0.05 after correction for multiple comparisons (see text for details). * Above a column (panel (b), (c) and (d)) denotes a change in contrast, whilst a * above connecting lines (panel (a), (c) and (d)) denotes a difference between groups or conditions. DLPFC: dorsolateral prefrontal cortex; L: left; lOFC: lateral orbitofrontal cortex; mOFC: medial orbitofrontal cortex; PPC: posterior parietal cortex; R: right; VLPFC: ventrolateral prefrontal cortex.

There were significant main effects of dimensional shift (ED vs ID) and target change (contingency change vs set change) on mean number of errors made (Figure 3(a)). More specifically, healthy participants made more errors when the rule change required an ED shift compared to an ID shift (*F* = 6.43, *p* = 0.014), and when the rule change occurred following a contingency change compared to a set change (*F* = 13.70, *p* < 0.001).

#### Patients with schizophrenia on placebo

There were no significant main effects of dimensional shift (*F* = 0.91, *p* = 0.347) or target change (*F* = 1.26, *p* = 0.270) on mean number of errors made (Figure 3(a)). Post hoc analysis (paired *t*-test, Bonferroni-corrected 0.05/2 = *p* < 0.025) revealed similar number of errors were made for ED vs ID shifts (*t* = 1.25, *p* = 0.242) and contingency change vs set change (*t* = 2.20, *p* = 0.056; Figure 3(a)).

#### Healthy comparison group vs patients with schizophrenia on placebo

Patients with schizophrenia on placebo made significantly more total errors than the healthy comparison group (*F* = 34.70, *p* < 0.001). Further analysis using four independent one-way ANOVAs revealed that patients with schizophrenia, compared to the healthy comparison group, made significantly more errors across all four categories; ID shift (*F* = 8.56, *p* = 0.017), ED shift (*F* = 25.30, *p* < 0.001), set change (*F* = 14.70, *p* = 0.004) and contingency change (*F* = 18.20, *p* = 0.002; Figure 3(a)). However, in the GLM there was no significant group effect on number of errors made for dimensional shift (*F* = 0.89, *p* = 0.347) or target change (*F* = 1.03, *p* = 0.312), meaning the patients with schizophrenia made a similar ratio of errors for ED vs ID shifting and contingency vs set change as the healthy comparison group.

#### Patients with schizophrenia on placebo, 100 µg and 250 µg roflumilast

**Figure 4.**
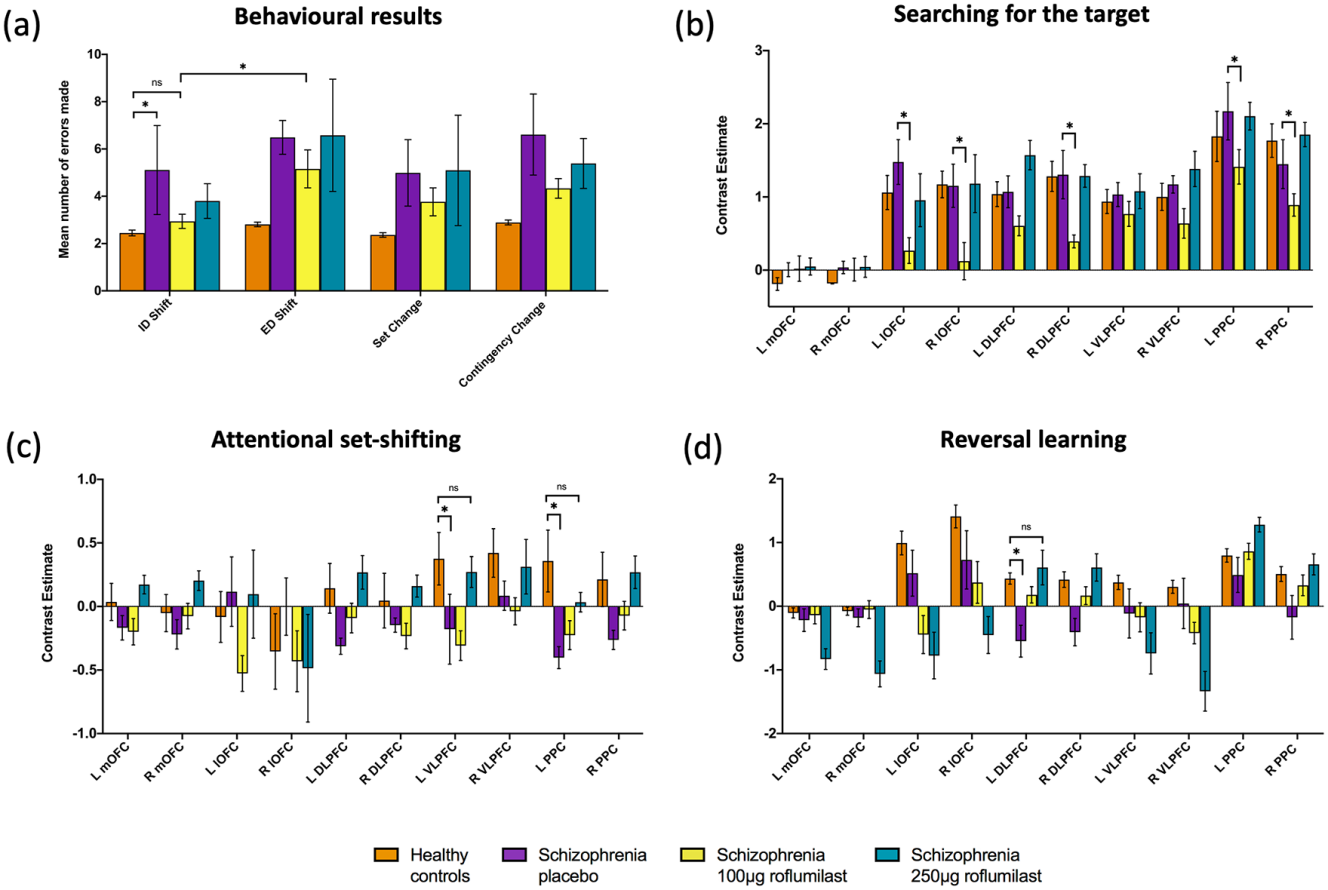
Behavioural and imaging results for the healthy comparison group and patients with schizophrenia on placebo, 100 μg and 250 μg roflumilast: (a) bar chart showing mean number of errors made whilst searching for the target according to category type of dimensional shift (intradimensional (ID) and extradimensional (ED)) and target change (set change and contingency change); (b) bar chart showing beta values extracted from the 10 regions of interest (ROIs) for the searching > known contrast; (c) bar chart showing beta values extracted from the 10 ROIs for the ED shift > ID shift contrast; (d) bar chart showing beta values extracted from the 10 ROIs for the contingency change > set change contrast. Error bars represent standard error of the mean (SEM) and * denotes *p* < 0.05 after corrections for multiple comparisons. * Above a column (panel (b), (c) and (d)) denotes a change in contrast, whilst a * above connecting lines (panel (a), (c) and (d)) denotes a difference between groups or conditions, and ‘ns’ denotes ‘no significance’. DLPFC: dorsolateral prefrontal cortex; L: left; lOFC: lateral orbitofrontal cortex; mOFC: medial orbitofrontal cortex; PPC: posterior parietal cortex; R: right; VLPFC: ventrolateral prefrontal cortex.

There was no significant drug effect on mean number of errors made for dimensional shift (*F* = 0.39, *p* = 0.667) or target change (*F* = 1.41, *p* = 0.251). However, grouped across all three drug conditions, there was a significant main effect of dimensional shift (*F* = 16.63, *p* < 0.001) on mean number of errors made, attributable to the 100 µg (*F* = 5.39, *p* = 0.026) not 250 µg roflumilast (*F* = 2.55, *p* = 0.119; Figure 4(a)) treatment. Post-hoc analysis (paired *t*-test, Bonferroni-corrected, 0.05/2 = *p* < 0.025) revealed the dimensional shift effect appeared to be due to a reduction in errors during ID shifts (although not formally significant, *t* = 2.14, *p* = 0.030), with ID errors reduced enough such that they were no longer statistically different from the healthy comparison group (*t* = 1.41, *p* = 0.172; Figure 4(a)). Further analysis of total task errors using a paired *t*-test revealed patients made significantly less errors on 100 µg roflumilast compared to placebo (*t* = 2.00, *p* = 0.038).

### Imaging results

All *p* values (except those reported from the repeated measures ANOVA) have been FDR-corrected with a false discovery rate of 0.1.

#### Searching for the target

In the healthy comparison group, there was significant bilateral activation in the lateral OFC (left *t* = 3.47, *p* = 0.002; right *t* = 3.96, *p* < 0.001), DLPFC (left *t* = 4.79, *p* < 0.001; right *t* = 3.87, *p* = 0.001), VLPFC (left *t* = 5.74, *p* < 0.001; right *t* = 8.45, *p* < 0.001) and PPC (left *t* = 4.66, *p* < 0.001; right *t* = 5.30, *p* < 0.001), with lower activity of the bilateral medial OFC (left *t* = –2.01, *p* = 0.031; right *t* = –2.14, *p* = 0.026). Patients with schizophrenia on placebo showed significant activation in the bilateral lateral OFC (left *t* = 3.69, *p* = 0.040; right *t* = 2.97, *p* = 0.011), DLPFC (left *t* = 4.88, *p* = 0.001; right *t* = 4.08, *p* = 0.003), VLPFC (left *t* = 2.95, *p* = 0.010; right *t* =  4.60, *p* = 0.002) and PPC (left *t* = 7.55, *p* < 0.001; right *t* = 5.05, *p* = 0.002), and activity did not vary significantly between patients on placebo and the healthy comparison group across any ROIs whilst searching for the target (*X*2 = 1.51, *p* = 0.219; Figure 3(b)).

**Figure 5.**
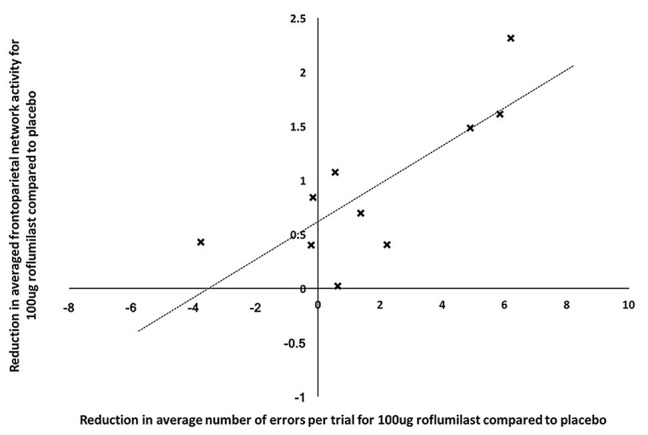
Scatter plot showing a correlation between reduction in average number of errors per trial and reduction in averaged network activity for 100 μg roflumilast compared to placebo.

For the patients with schizophrenia across the three drug conditions there was a significant main effect of ROI (*F* = 4.23, *p* < 0.001) and drug (*F* = 5.30, *p* = 0.019) whilst searching for the target. There was also a significant three-way interaction between ROI, drug and drug order (*F* = 2.06, *p* = 0.003). Post hoc analysis revealed that 100 µg roflumilast significantly reduced activity in the bilateral OFC (left *t* = 2.85, *p* = 0.038; right *t* = 1.88, *p* = 0.046), bilateral PPC (left *t* = 2.64, *p* = 0.027; right *t* = 1.87, *p* = 0.037) and right DLPFC (*t* = 2.54, *p* = 0.021) compared to placebo (Figure 4(b)). A further correlation analysis revealed that, for 100 µg dose compared to placebo, there was a positive correlation between the reduction in overall network activity and the number of errors made (*r* = 0.782, *p* = 0.008; Figure 5).

#### Attentional set-shifting

In the healthy comparison group, the left VLPFC and left PPC were activated, but following FDR-correction the left VLPFC was no longer significant (*t* = 2.09, *p* = 0.088), while the left PPC remained significant (*t* = 1.45, *p* = 0.046). In patients with schizophrenia on placebo, the left DLPFC (*t* = –2.45, *p* = 0.037) and left PPC (*t* = –2.31, *p* = 0.045) had reduced activity, although these did not survive FDR-correction (*p* = 0.148 and *p* = 0.091, respectively).

When comparing patients with schizophrenia on placebo to the healthy comparison group there was a main group effect on activity (*X*2 = 12.7, *p* < 0.001). Post hoc analysis revealed the left PPC (*U* = 35, *p* = 0.029) and left VLPFC (*U* = 43, *p* = 0.048) were significantly reduced in activity in the patient group when engaging in an ED shift compared to an ID shift (Figure 3(c)). For the patients with schizophrenia across the three drug conditions there were no significant main effects of ROI (*F* = 1.04, *p* = 0.418), drug (*F* = 0.44, *p* = 0.653), drug order (*F* = 1.58, *p* = 0.272) or interaction whilst shifting attentional sets (Figure 4(c)). However, further analysis using paired *t*-tests revealed that 250 µg roflumilast appeared to recover a deficit in left VLPFC and left PPC such that they were no longer statistically different to the healthy comparison group (*t* = 0.32, *p* = 0.755 and *t* = 0.93, *p* = 0.360, respectively).

#### Reversal learning

In the healthy comparison group, there was significant activation of the bilateral lateral OFC (left *t* = 2.66, *p* = 0.022; right *t* = 3.92, *p* = 0.004), the left DLPFC (*t* = 2.47, *p* = 0.024) and the bilateral PPC (left *t* = 3.78, *p* = 0.003; right *t* =  2.17, *p* = 0.035). In patients with schizophrenia on placebo, no ROI activated for the contrast following FDR-correction, although the left DLPFC (*U* = 44, *p* = 0.027) showed significantly reduced activity compared to the healthy comparison group while engaging in reversal learning following a contingency change compared to a set change (Figure 3(d)).

For the patients with schizophrenia across the three drug conditions there was a significant interaction of ROI and drug (*F* = 2.33, *p* = 0.005). However, further analysis using paired *t*-tests revealed there was no significant difference in activation of any of the ROIs across each of the drug conditions, suggesting it could be an ROI effect at different doses (Figure 4(d)). We note that the difference between patients with schizophrenia on placebo and the healthy comparison group in the left DLPFC was no longer significant after 250 µg of roflumilast (*t* = 0.37, *p* = 0.712).

## Discussion

This study used an fMRI-optimised version of the ID/ED attentional set-shifting task (Hampshire and Owen, 2006) to investigate the ability of the PDE4 inhibitor, roflumilast, to improve attentional set-shifting in patients with schizophrenia. First, we replicated the expected pattern of behavioural performance and most of the associated imaging findings in healthy participants, as individuals made more errors when shifting attentional set and engaging in reversal learning. Conversely, individuals with schizophrenia demonstrated a widespread deficit in task performance, suggesting difficulties in forming, maintaining, using and/or shifting attentional sets. The patient group showed preserved activity across frontoparietal areas while searching for the target, but demonstrated a deficit in brain activation during attentional set-shifting (left VLPFC and left PPC) and reversal learning (left DLPFC). The 100 µg dose of roflumilast improved formation of attentional sets and reduced activity across the network of ROIs while searching for the target, while the 250 µg dose appeared to normalise deficits in ROI activity during attentional set-shifting and reversal learning.

### Replication of previous findings in the healthy comparison group

Our behavioural data replicated the increased cognitive cost of shifting attentional set and of engaging in reversal learning as healthy participants took more trials to solve these rule changes (Hampshire and Owen, 2006; Williams-Gray et al., 2008). Attentional set-shifting was associated with activation of the left VLPFC (although this did not survive correction for multiple comparisons) and left PPC as found previously (Asari et al., 2005; Hampshire and Owen, 2006), while reversal learning was accompanied by expected bilateral lateral OFC activation (Hampshire and Owen, 2006; Williams-Gray et al., 2008), as well as the left DLPFC and bilateral PPC. The residual activity of the left DLPFC may be related to the increased working memory component of a contingency change compared to a set change (Barbey et al., 2013), while the PPC may be involved due to its role in coordination of executive control, as found previously (Hampshire and Owen, 2006).

### Investigation of deficits in cognitive flexibility in patients with schizophrenia

Consistent with previous studies there were impairments in attentional set-shifting task performance in this patient group. The ROI activity for the searching contrast was surprisingly similar to the healthy comparison group, given patients made more errors on the task. While not formally tested in this study, our findings of impaired performance with preserved activity across the dorsal frontoparietal attention network supports an emerging theory that patients with schizophrenia have an abnormally narrow but intense focusing of attention that inhibits accurate distribution of resources across cognitively demanding tasks (Luck et al., 2019). In this study, the similar levels of activation observed may therefore indicate that although the patients are engaging the dorsal frontoparietal attention network, they do so inefficiently, leading to impaired performance.

Unlike the healthy comparison group who demonstrated an increased cognitive cost of ED shifting, the patients’ errors were not significantly increased following an ED shift compared to an ID shift. This may explain why no significant ROI activity was observed for this contrast, as shifting both within and between dimensions may have similar high processing requirements. This could be caused by an inability to form (Pantelis et al., 1999), maintain (Pantelis et al., 2004) or effectively use (Ceaser et al., 2008) an attentional set, which is required for them to shift more efficiently within dimensions (ID) than between dimensions (ED). This broad deficit is further supported by findings that patients with schizophrenia made significantly more errors for both ID and ED shifting compared to the healthy comparison group. Importantly, the patients did not recruit the left PPC and VLPFC while shifting attentional set, which were both represented in the healthy comparison group activations and in previous literature (Asari et al., 2005; Hampshire and Owen, 2006).

Similar to the ED vs ID shifting and unlike the healthy comparison group, the patients made the same number of errors following a contingency change as a set change, which may be due to a failure to engage in reversal learning and activate the lateral OFC. Again, similar to attentional shifting, patients with schizophrenia made significantly more errors for both contingency change and set change compared to the healthy comparison group, suggesting a broader deficit beyond reversal learning that might include associative learning. While some studies highlight only a reversal learning *deficit* in patients with schizophrenia (Culbreth et al., 2016; Waltz and Gold, 2007), it is interesting to compare our behavioural results with those of Reddy et al. (2016), who found that patients made just as many errors during discrimination learning as during reversals. Therefore, previously described deficits in reinforcement learning (Murray et al., 2008) cannot be ruled out as a contributor towards the broad deficit in this study. Theoretically, if the patients struggled to form associations, then learning new associations would be just as difficult following a set change as a contingency change. Furthermore, this would be predicted to lead to weak stimulus-contingency associations and thus engagement of areas involved in reversal learning would be limited, as seen with the lateral OFC. Additionally, the patients show significantly reduced activity in the left DLPFC compared to the healthy comparison group, a region which may help manage the working memory component of learning and switching associations (Barbey et al., 2013).

It is important to note there was a significant age difference between the healthy comparison group (mean ± standard deviation (SD) = 57.67 ± 8.32) and patients with schizophrenia (mean ± SD = 37.4 ± 7.9), as the healthy comparison group was taken from a previously collected dataset of older cognitively normal adults. Older individuals (mean ± SD = 57.8 ± 1.1) tend to perform poorer than younger cognitively normal individuals (mean ± SD = 34.2 ± 10), but only at the ED shifting stage (Robbins et al., 1998). Thus, the deficit observed in the patient group compared to the controls is the opposite of an ageing effect. As the age differed between groups, we were not able to include age as a covariate in our analyses. Consequently, we explored the relationship of age and ROI activity by performing multiple Pearson’s correlations between ROI activity and age across all of the ROIs in each of the contrasts, for which there were no significant differences.

### Testing the translation of roflumilast to improve attentional set-shifting deficits in schizophrenia

In patients with schizophrenia, only the 100 µg dose of roflumilast significantly reduced the number of errors, which correlated with reduced activity during searching across several ROIs. Secondly, under this dose, patients displayed the expected increased cognitive cost of attentional set-shifting (ED errors > ID errors), due to fewer ID errors compared to ED errors. In fact, the drug reduced ID errors to the extent that they were no longer statistically different from those of the healthy comparison group. This suggests the lower dose of the drug helps patients form, maintain and/or use attentional sets, but does not improve deficits in shifting between these attentional sets. Roflumilast inhibits the breakdown of cAMP, and this in turn enhances the effect of excitatory neurotransmission, including neurotransmitter actions relevant for PFC-dependent cognition (Schoffelmeer et al., 1985). The cognitive enhancing effect of only the lower dose aligns with research showing cognitive performance and dopamine levels in the PFC tend to follow an inverted U-shape, such that too little or too much dopamine impairs performance (Cools et al., 2011). Additionally, previous studies testing roflumilast’s cognitive enhancing properties have also reported optimum behavioural performance improvement at the 100 µg dose in sensory gating (Heckman et al., 2018) and episodic memory in young (Van Duinen et al., 2018) and older humans (Blokland et al., 2019).

Our findings appear to differ with previous studies showing that improved behavioural performance at 100 µg is associated with *increased* functional activity in related brain regions (Blokland et al., 2019; Heckman et al., 2018; Van Duinen et al., 2018). This may be due to different processes requiring a different optimum level of neural activity in these brain regions. Alternatively, it could be due to differences in drug exposure. In the previous studies, only a single dose of roflumilast was given, so the most likely mechanism for an effect was an increase in excitatory neurotransmission (Kuroiwa et al., 2012). However, in this current study, the drug was given for eight consecutive days, potentially allowing more long-term cellular changes such as long-term potentiation (LTP) in the PFC (Selemon, 2013). cAMP activates protein kinase A (PKA), which in turn phosphorylates cAMP response element-binding protein (CREB), an activated transcription factor that leads to the insertion of new membrane receptors (Song et al., 2013). Additionally, the dopamine and cAMP-regulated phosphoprotein of molecular weight 32 kDa (DARPP-32) is involved in transcriptional and behavioural effects of dopamine (Svenningsson et al., 2004). Importantly, improvement in cognitive tasks has been associated with the phosphorylation of both CREB and DARPP-32 in as little as 4 h after treatment with a dopamine agonist (Hotte et al., 2006). Therefore, direct inhibition of PDE4 by roflumilast might lead to increased ionotropic α-amino-3-hydroxy-5-methyl-4-isoxazolepropionic acid (AMPA) receptor insertion. In order to fit with our findings, this would need to lead to more efficient circuitry, represented by reduced BOLD activity and an improvement in behavioural performance. This theory is supported by previous findings, as roflumilast has been shown to improve verbal memory in patients with schizophrenia that was accompanied by a numerical reduction of bilateral DLPFC activation (Gilleen et al., 2018).

In contrast to the 100 µg dose, 250 µg roflumilast exhibited the largest drug effect on ROI activity during attentional set-shifting and reversal learning. These changes were observed primarily across the VLPFC, DLPFC and PPC. The changes fit with a normalisation or attenuation of impaired activity of these regions on placebo when compared to the healthy comparison group while shifting attentional sets. While the within-subject drug effect did not survive multiple comparisons correction, the loss of difference with the healthy comparison group informs us of the drug’s potential. A larger sample size is required in order to demonstrate a robust drug effect. Additionally, roflumilast demonstrated a dose-dependent effect on ROI activity for these contrasts (although this did not remain significant after correction for multiple comparisons), compared to the searching contrast where the lower dose was more effective. This might be related to the understanding that different cognitive tasks have different dopamine levels for optimum function (Cools et al., 2011). Therefore, whilst 100 ug of roflumilast might have led to the optimum dopamine levels for the searching contrast, the 250 ug dose might not have been high enough to produce optimum dopamine levels for attentional set-shifting and reversal learning. The findings across both doses provide preliminary support for the hypothesised role of PDE4 inhibitors in set-shifting tasks, as seen in rodent studies, but demonstrate that the precise effects may differ by dose.

### Future research

Future research should first aim to rectify limitations of this study in a replication study, by including a larger cohort of participants, as well as using a randomised stimuli order and increasing the time between visits to minimise the drug order effect on task performance. Finally, there may also be limitations with the task itself, particularly in this clinical population. The widespread impaired performance across each of the four categories (ID shifts, ED shifts, set change and contingency change) suggests that some patients may have struggled to form, maintain or use attentional sets and reward-contingency associations. This may require further training on a novel, simplified set-shifting task, increasing the number of trials and participants that can be utilised in the analysis. Indeed, one reason for the failure to observe an improvement in attentional set-shifting behaviour after roflumilast may be due to difficulty in forming an attentional set in the first place.

## Conclusions

In summary, we replicated the performance of healthy participants on this cognitive flexibility task, along with some, but not all, of the neuroimaging findings. Patients with schizophrenia showed a broad deficit across every aspect of this task, which may be due to a deficit in forming, maintaining or using attentional sets and reward-contingency associations. Roflumilast modulated performance and brain activity measured with fMRI and suggested a potential dose-dependent effect, with 100 µg improving behavioural performance in formation of attentional sets, while 250 µg normalised deficits in activity in brain regions associated with attentional set-shifting and reversal learning. This study provides preliminary support for the ability of roflumilast to improve cognitive flexibility deficits in patients with schizophrenia, and studies with larger populations and additional set-shifting tasks are now required.
